# HO‐1 in lymph node metastasis predicted overall survival in patients with esophageal squamous cell carcinoma receiving neoadjuvant chemoradiation therapy

**DOI:** 10.1002/cnr2.1477

**Published:** 2021-07-15

**Authors:** Ryujiro Akaishi, Fumiyoshi Fujishima, Hirotaka Ishida, Junichi Tsunokake, Takuro Yamauchi, Yusuke Gokon, Shunsuke Ueki, Toshiaki Fukutomi, Hiroshi Okamoto, Kai Takaya, Chiaki Sato, Yusuke Taniyama, Tomohiro Nakamura, Naoki Nakaya, Takashi Kamei, Hironobu Sasano

**Affiliations:** ^1^ Department of Surgery Tohoku University Graduate School of Medicine Sendai Japan; ^2^ Department of Pathology Tohoku University Hospital Sendai Japan; ^3^ Department of Health Record Informatics Information Security Tohoku Medical Megabank Organization, Tohoku University Sendai Japan; ^4^ Department of Health and Social Services Saitama Prefectural University Graduate School Koshigaya Japan

**Keywords:** chemotherapy, metastases, pathology, radiation therapy

## Abstract

**Background:**

Lymph node metastasis is one of the pivotal factors of the clinical outcomes of patients with esophageal cancer receiving neoadjuvant chemoradiation therapy (NACRT). Both the nuclear factor‐erythroid 2‐related factor 2 (Nrf2) signaling pathway and heme oxygenase‐1 (HO‐1) are frequently upregulated in various human malignancies and associated with resistance to chemoradiation therapy, subsequently resulting in adverse clinical outcomes. However, the Nrf2 and HO‐1 status in lymph node metastasis and their differences between primary and metastatic lesions are unknown.

**Aims:**

To examine the levels of Nrf2 signaling proteins and HO‐1 in primary and metastatic lesions of patients with esophageal squamous cell carcinoma using immunohistochemistry.

**Methods and Results:**

We immunolocalized Nrf2 signaling proteins in 69 patients with lymph node metastases, who received NACRT with 5‐fluorouracil and cisplatin before esophagectomy. We also compared the findings between primary and metastatic lesions. Residual lymph node metastases were detected in 30 patients and among them, both primary and metastatic lesions were available for evaluation in 25 patients. Subsequently, we correlated the results with patients' survival. Nrf2, HO‐1, and the Ki‐67 labeling index were all significantly lower in the patients with lymph node metastases than in those with primary tumors. Carcinoma cells with high HO‐1 levels were significantly associated with pathological resistance to NACRT. These results suggested that overall and disease‐free survival of esophageal squamous cell carcinoma were significantly associated with both pN2 and high HO‐1 levels, respectively.

**Conclusions:**

Protein expression in the Nrf2 pathway was significantly lower in patients with lymph node metastases than in those with primary lesions. HO‐1 levels in lymph node metastases could be used to predict the eventual clinical outcome of patients with esophageal cancer receiving NACRT.

## 1 INTRODUCTION

Esophageal cancer is one of the most lethal gastrointestinal malignancies.^1^ Esophageal squamous cell carcinoma (ESCC) is the most frequent type in Japan and other East Asian countries. The standard therapy for locally advanced ESCC is radical resection with extensive lymph node (LN) dissection following neoadjuvant chemotherapy (NAC).^2^ Radiation in conjunction with neoadjuvant chemoradiation (NACRT) is an alternative treatment for locally advanced ESCC.^3^, ^4^, ^5^

LN metastasis is a well‐known adverse prognostic factor in ESCC, especially for patients receiving NAC or NACRT.^6^, ^7^ The histopathological tumor regression grade (TRG) in LN metastases predicts clinical outcomes of patients receiving NAC.^8^, ^9^ However, TRG has been evaluated mostly in primary tumors.^10^ In addition, there are no standard methods for evaluating the histological efficacy of preoperative therapy in LN metastases.

The nuclear factor‐erythroid 2‐related factor 2 (Nrf2) signaling pathway plays key roles in regulating antioxidative protein expression.^11^ Among those proteins, heme oxygenase 1 (HO‐1) exerts antioxidant and apoptotic effects, facilitating carcinoma cell proliferation and resistance to anti‐tumor therapy.^12^ In addition, both Nrf2 and HO‐1 are frequently up‐regulated and correlated with tumor progression, aggravation, resistance to treatment, and adverse clinical outcomes in various human malignancies.^13^, ^14^, ^15^, ^16^, ^17^ In ESCC, Nrf2 over‐expression in primary tumors is correlated with NACRT resistance and adverse clinical outcomes.^18^, ^19^ However, the status of Nrf2 in LN metastases in patients receiving NACRT and the differences in Nrf2 between primary and LN metastatic lesions are unknown.

Therefore, we intended to clarify the following: (1) the status of antioxidant proteins in LN metastases of patients with ESCC receiving NACRT and its correlation with histological TRG, (2) the differences of antioxidant proteins between primary tumors and metastases, and (3) the correlation of the findings with residual LN metastases following NACRT in patients with ESCC.

## 2 METHODS

### 2.1 ESCC cases

We examined 69 patients who were diagnosed with stage II–IV ESCC between 2011 and 2015 at the Tohoku University Hospital, Sendai, Japan. All the patients in this study had received NACRT before esophagectomy with regional LN dissection. Residual primary tumors were identified by histological examination in surgical specimens of 50/69 patients. Of the 50 patients, residual LN metastases were detected by histological examination in 30 patients. Five cases with no residual LN metastatic lesions seen during an extensive pathological evaluation were excluded from this study.

Response Evaluation Criteria in Solid Tumors (RECIST), version 1.1 was used to evaluate the clinical therapeutic effects of NACRT.^20^ The eighth edition of the American Joint Committee on Cancer/Union for International Cancer Control Tumor‐Node‐Metastasis (TNM) staging system for esophageal carcinoma was used for tumor staging.^21^ The overall survival (OS) and disease‐free survival (DFS) rates were determined as the duration from the initial surgery to demise or cancer recurrence, respectively, or last censoring.

### 2.2 Ethics approval and consent to participate

The study protocol was approved by the Ethics Committee of the Tohoku University School of Medicine (Accession No. 2020‐1‐87), and informed consent was obtained from all patients prior to surgery through signed consent forms.

### 2.3 NACRT and surgery

The patients were administered a continuous intravenous infusion of 5‐fluorouracil (400 mg/m^2^/day) for 24 h on days 1–5 and 8–12, and cisplatin (40 mg/m^2^) for 2 h on days 1 and 8. They also received radiotherapy (total of 30 Gy in 15 fractions over 3 weeks). The radiation field was long and T‐shaped and contained supraclavicular and mediastinal LNs in the cases of cervical or upper and middle thoracic tumors, and perigastric LNs in those with lower thoracic tumors. In patients who underwent thoracoscopic esophageal subtotal excision, gastric tube reconstruction was performed by hand‐assisted laparoscopy or open laparotomy, and cervical esophagogastric anastomosis with regional LN dissection were performed subsequently.

### 2.4 Evaluation of therapeutic response

The histopathological findings of the resected specimens, including the invasive length of the primary lesions, status of LN metastases, and histological differentiation, were examined independently by two of the authors (F.F. and H.S.). Dissected regional LNs were classified as non‐metastasized LNs or plausible positive metastatic LNs (pp‐MLNs). The histological TRG of the primary tumor and each pp‐MLN were classified tentatively as follows: Grade 0, ineffective—neither coagulative necrosis nor cellular/structural changes were identified in the lesion; Grade 1a: coagulative necrosis or histopathological disappearance of the tumor in ≤one‐third of the whole lesion; Grade 1b: coagulative necrosis or histological disappearance of the tumor in one‐third to two‐third of the whole lesion; Grade 2: coagulative necrosis or disappearance of carcinoma cells in >two‐third of the entire lesion, although histopathologically defined viable tumor cells were still discernible; Grade 3: entire lesion displaying coagulative necrosis and/or replaced by interstitial fibrosis, and no histologically identified viable carcinoma cells.^10^ Grades 0 and 1 were considered “ineffective” and Grades 2 and 3 “effective.”^18^, ^19^

The ratio of “effective” LN cases to all pp‐MLNs was calculated and defined as the chemoradiation therapy effective rate (CRER). The CRER allowed the classification of individual patients into either the low‐CRER (LCRER, ≥0% and <50%) group or high CRER (HCRER, ≥50%) group.^8^ LCRER was considered “ineffective” and HCRER “effective.”^8^

### 2.5 Immunohistochemistry

Metastatic LNs with the largest volume of residual tumors were selected after a careful histological review. Tissues had been fixed in neutral 10% formalin and embedded in paraffin wax. Details of immunostaining, including primary antibodies, methods of antigen retrieval, and buffers used, are summarized in Table S1. A product of oxidative DNA damage caused by hydroxyl radicals, 8‐hydroxydeoxyguanosine (8‐OHdG), was used to evaluate the levels of reactive oxygen species (ROS) in carcinoma cells.^22^, ^23^

Briefly, 4 μm‐thick tissue sections were mounted on clean glass slides and deparaffinized with xylene and ethanol. Sections were then heated for antigen retrieval, as described in Table S1. To inhibit non‐specific antibody binding, the treated sections were incubated with 10% normal rabbit serum (Histofine Kit; Nichirei Bioscience, Tokyo, Japan) for Nrf2 and 8‐OHdG, or 10% normal goat serum (Histofine Kit; Nichirei Bioscience) for HO‐1, at 24°C for 30 min. The sections were then incubated with primary antibodies overnight at 4°C. Regarding 8‐OHdG immunohistochemistry, H_2_O_2_ processing was performed following overnight treatment with primary antibodies to inhibit the reaction between the primary antibodies and 8‐OHdG from the direct reaction between hydroxyl radicals generated from H_2_O_2_ and normal deoxyguanosine from the tissues. In addition, endogenous peroxidase activity was suppressed by immersing the tissue sections in 0.3% H_2_O_2_ for 30 min at 24°C. The sections were subsequently incubated with biotinylated rabbit anti‐mouse or goat anti‐rabbit immunoglobulin (Histofine Kit; Nichirei Bioscience) and peroxidase‐labeled streptavidin (Histofine Kit; Nichirei Bioscience) at 24°C for 30 min. The antigen–antibody complexes were then visualized using 1.0 mmol/L of 3.3‐diaminobenzidine in 50 mol/L of Tris–HCl buffer (pH 7.6) containing 0.006% H_2_O_2_ and were subsequently stained with hematoxylin. Ki‐67 immunostaining was performed according to the manufacturer's instructions (EnVision FLEX Kit High pH; Agilent Technologies, Santa Clara, CA).

Immunoreactivity were all independently evaluated by two of the authors (R.A. and F.F.) who were blinded to the clinicopathological parameters of all patients. Both Nrf2 and Ki‐67 were examined in the nuclei of carcinoma cells, whereas HO‐1 and 8‐OHdG were assessed in the cytoplasm of carcinoma cells. In addition, 8‐OHdG, which is an oxidative stress marker was also used to study ROS levels in carcinoma cells.^22^, ^23^ Nrf2, HO‐1, and 8‐OHdG were all semi‐quantitatively assessed using the H‐score, which represents the ratio of immuno‐positive cells to the overall carcinoma cells in the examined lymph nodes, and multiplied by the relative immunointensity (0, negative; 1, weak; 2, moderate; 3, marked), ranging from 0 to 300.^24^, ^25^ Nuclear Ki‐67 immunoreactivity was scored by counting the proportion of immuno‐positive cells.^13^ We tentatively determined the optimal cut‐off values of the histological response of LN metastases using a receiver operating characteristic (ROC) curve.^24^ These cut‐off values included Nrf2, 95; HO‐1, 78; 8‐OHdG, 85; and Ki‐67, 38%. The threshold of HO‐1 in primary tumors was 125. Cases with an H‐score or a Ki‐67 labeling index below the cut‐off were considered as having “low expression,” while those with scores greater than the cut‐off value as having “high expression.” Stromal cells and lymphocytes were used as positive controls during immunohistochemistry.^26^

### 2.6 Statistical evaluation

All statistical analyses were performed using JMP Pro version 14.2.0 (SAS Institute, Cary, NC). Continuous data were analyzed with Student's *t*‐test or the Wilcoxon rank sum test. Correlations between two variables were assessed using the Pearson Chi‐squared test, Fisher's exact test, or the Wilcoxon rank sum test, when appropriate. OS and DFS curves were generated using the Kaplan–Meier method and compared with the log‐rank test. A Cox proportional hazard model was used for both univariable and multivariable analyses. The goodness of fit of the Cox proportional hazard model was evaluated using the likelihood ratio test in this analysis. Paired values were compared using the Wilcoxon signed rank test. *P* values <.05 were considered statistically significant.

## 3 RESULTS

### 3.1 Post‐NACRT histological TRG and antioxidant proteins in LN metastases

**FIGURE 1:**
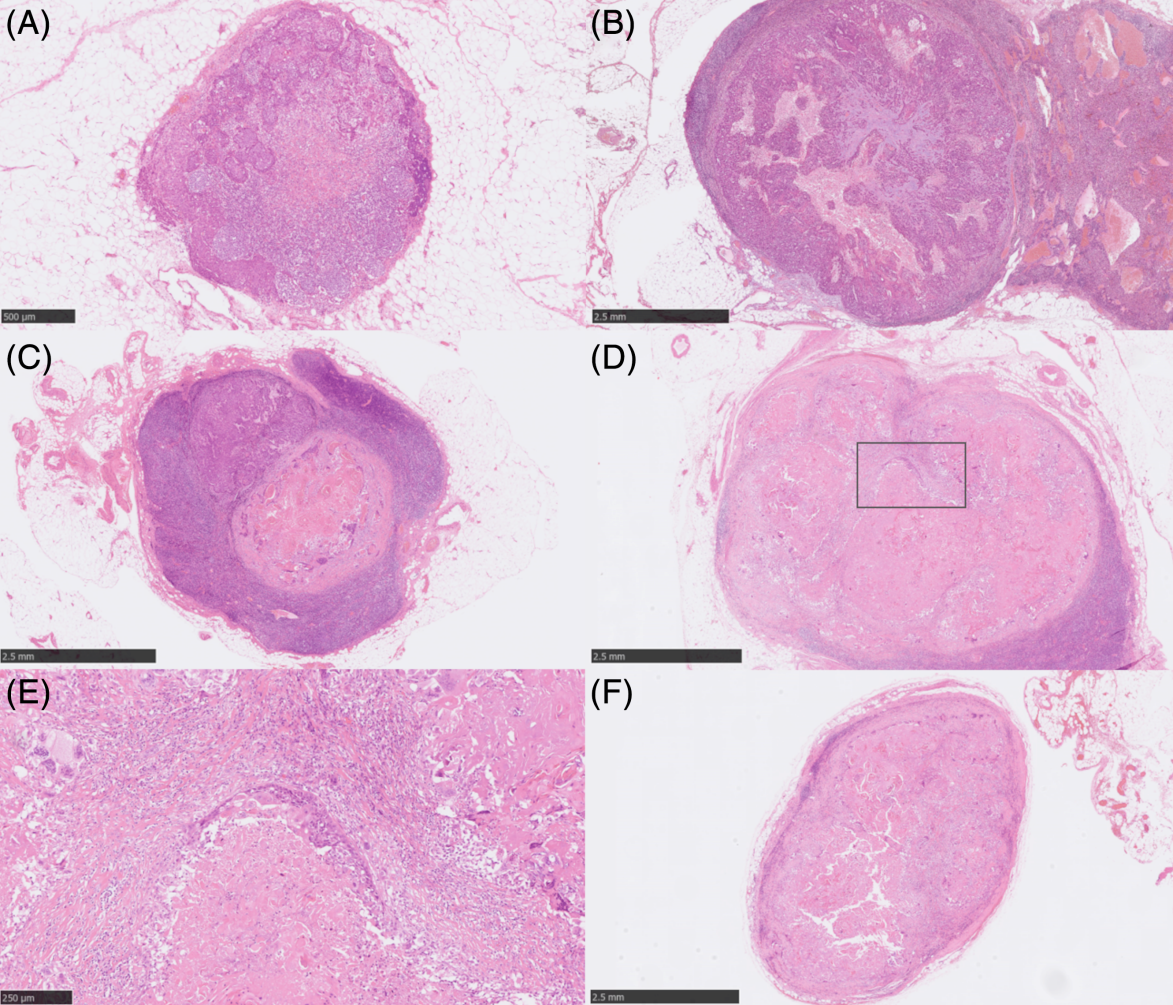
Representative illustrations of the histological tumor regression grades of lymph node metastases. (A) Grade 0: neither coagulative necrosis nor cellular or structural alterations were detected in all the lesions. (B) Grade 1a: coagulative necrosis or histological disappearance of the carcinoma cells present in ≤one‐third of the whole tumor. (C) Grade 1b: coagulative necrosis or histological disappearance of the carcinoma cells in one‐ to two‐third of the whole tumor. (D) Grade 2: coagulative necrosis or disappearance of carcinoma cells in >two‐third of the whole tumor, but histologically viable carcinoma cells were still identified. (E) Enlarged image of the squared area in (D) with viable tumor cells. (F) Grade 3: the whole tumor was composed of coagulative necrosis and/or replaced by interstitial fibrosis, with or without granulomatous tissue reaction, with no histologically detected viable carcinoma cells

**FIGURE 2:**
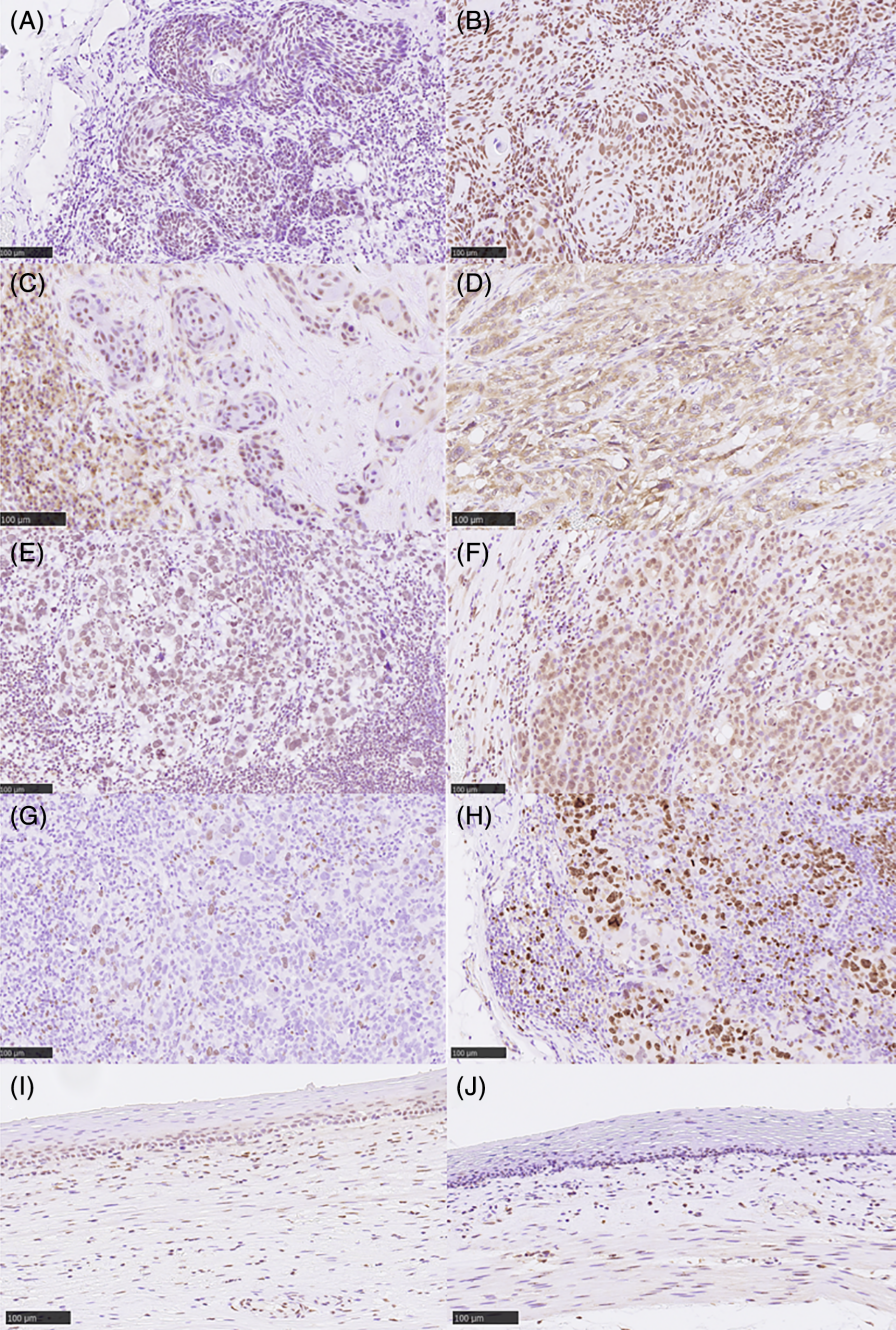
Representative illustrations of immunohistochemistry. (A) Low Nrf2 immunoreactivity. (B) High Nrf2 nuclear immunoreactivity in carcinoma cells. (C) Low HO‐1 immunoreactivity. (D) High cytoplasmic HO‐1 immunoreactivity in carcinoma cells. (E) Low and (F) high cytoplasmic 8‐OHdG in carcinoma cells. (G) Low and (H) high nuclear Ki‐67 immunoreactivity in carcinoma cells. (I) Nrf2 immunoreactivity in non‐cancerous or non‐pathological esophageal epithelium. (J) HO‐1 immunoreactivity in non‐cancerous or non‐pathological esophageal epithelium

Representative micrographs of the histological TRG of LN metastases are shown in Figure 1. Multiple pp‐MLNs were detected in 64.0% (16/25) of the patients (Table S2). In addition, the TRG of pp‐MLNs varied in 75.0% (12/16) of those with multiple pp‐MLNs (Table S2). Among the ineffective group, 86.7% (13/15) were also classified as NACRT‐ineffective, based on the features of their primary tumors. However, 40% (4/10) of the effective group were re‐classified as NACRT‐ineffective (Table S3). Representative micrographs of Nrf2, HO‐1, 8‐OHdG, and Ki‐67 are shown in Figure 2. In the non‐cancerous esophageal squamous epithelium within the radiation field, the relative immuno‐intensity of Nrf2 and HO‐1 was weak, which made assessment of the findings difficult (Figure 2). Clinical resistance to NACRT was significantly associated with high HO‐1 expression (*P* = .028; Table 1).

**TABLE 1:** Expression status of the biomarkers and its correlation with clinicopathological features

		Nrf2 Low	Nrf2 High		HO‐1 Low	HO‐1 High		8‐OHdG Low	8‐OHdG High		Ki‐67 Low	Ki‐67 High	
Clinicopathological features	N	7	18	*P*	8	17	*P*	17	8	*P*	15	10	*P*
Age				.695			.194			.667			.442
≧65	10	2	8		5	5		6	4		5	5	
<65	15	5	10		3	12		11	4		10	5	
Gender				1.000			1.000			1.000			.150
Male	23	7	16		7	16		16	7		15	8	
Female	2	0	2		1	1		1	1		0	2	
pT^a^				.186			.295			.721			1.000
pT1	3	1	2		1	2		2	1		1	2	
pT2	11	4	7		4	4		6	2		6	2	
pT3	14	2	12		3	11		9	5		8	6	
pN^a^				1.000			1.000			.344			.667
pN1	17	5	12		6	11		12	5		11	6	
pN2	8	2	6		2	6		5	3		4	4	
Pathological stage^a^				.490			.569			1.000			1.000
Stage II	2	1	1		1	1		1	1		1	1	
Stage III	23	6	17		7	16		16	7		14	9	
Differentiation^a^				.291			.391			.267			.586
G1	4	1	3		1	3		4	0		2	2	
G2	12	2	10		3	9		9	3		7	5	
G3	9	4	5		4	5		6	3		6	3	
Microlymphatic invasion				.407			1.000			.389			1.000
Absence	11	2	9		3	8		6	5		7	4	
Presence	14	5	9		5	9		11	3		8	6	
Microvascular invasion				.407			1.000			1.000			1.000
Absence	11	2	9		4	7		7	4		7	4	
Presence	14	5	9		4	10		10	4		8	6	
Histological efficacy (Primary tumor)^b^				.640			.359			1.000			.088
Ineffective (Grade 0‐1b)	17	4	13		4	13		11	6		8	9	
Effective (Grade 2)	8	3	5		4	4		6	2		7	1	
Histological efficacy (LN metastasis)				.075			.028*			.088			.211
Ineffective (LCRER)	15	2	13		2	13		8	7		7	8	
Effective (HCRER)	10	5	5		6	4		9	1		8	2	

Abbreviations: HCRER, high chemoradiation therapy effective rate; LCRER, low chemoradiation therapy effective rate; LN, lymph node.

^a^ Tumor‐node‐metastasis (TNM) classification based on the 8th edition of the TNM classification of malignant tumors.

^b^ Histopathological features based on the Japanese Classification of Esophageal Cancer, 11th edition (Japan Esophageal Society 2015).

* Statistical significance.

### 3.2 Comparison of Nrf2, HO‐1, Ki‐67, and 8‐OHdG in primary esophageal tumors and LN metastases

**FIGURE 3:**
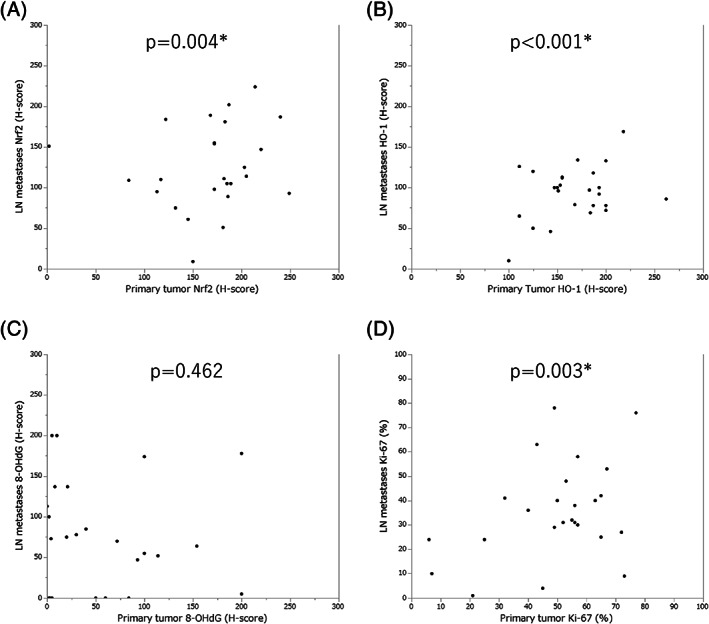
Scatter plots of H‐score and Ki‐67 labeling index in the specimens of primary tumors and lymph node (LN) metastases. Primary tumors and LN metastases were compared using the Wilcoxon signed rank test. Statistically significant differences were detected in (A) Nrf2 (*P* = .004), (B) HO‐1(*P* < .001), and (D) Ki‐67 (*P* = .003). (C) No significant difference was detected in 8‐OHdG, although the median 8‐OhdG H‐score was higher in LN metastases than in primary tumors (LN metastases: 70; primary lesions: 30)

The H‐scores of Nrf2 (*P* = .004), HO‐1 (*P* < .001), and the Ki‐67 labeling index (*P* = .003) were significantly lower in LN metastases than primary lesions (Figure 3). There were no significant differences in the H‐score of 8‐OHdG. However, its median value tended to be higher in LN metastases than primary tumors (LN metastases, 70; primary tumors, 30).

### 3.3 Overall survival and disease‐free survival analysis

**FIGURE 4:**
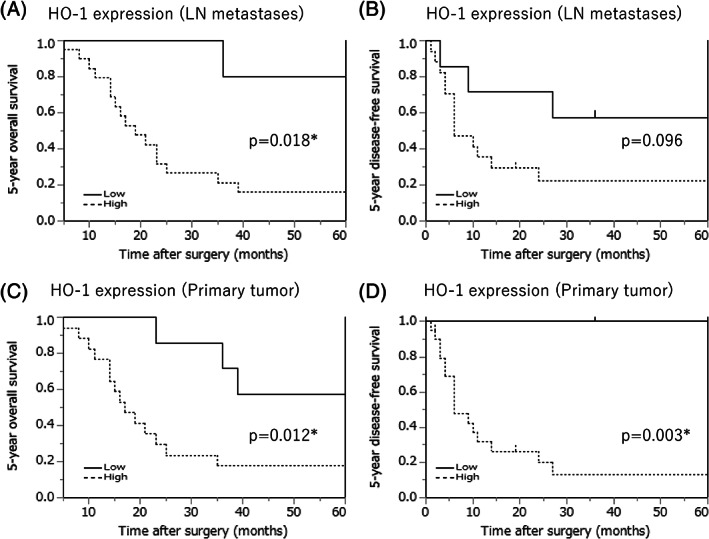
Kaplan–Meier curves of overall survival (OS) and disease free survival (DFS) of esophageal squamous cell carcinoma cases with lymph node (LN) metastases, classified according to HO‐1 levels in LN metastases and primary tumors. The 5‐year OS was significantly shorter in the high HO‐1 group (A). No significant difference was detected in the DFS analysis (B). Five‐year OS and DFS were also significantly shorter in the primary tumor group with high HO‐1 levels (C) and (D)

The results of the Kaplan–Meier analysis of patients with LN metastases, classified according to HO‐1 levels in LN metastases and primary tumors, are summarized in Figure 4. The 5‐year OS rate was significantly lower in patients with high HO‐1 levels (*P* = .018). In the primary tumor group, the 5‐year OS and DFS rate were significantly lower in patients with high HO‐1 levels than in those with low levels. However, there were no significant differences in the 5‐year DFS of the patients (Figure 4). In the univariable Cox analysis, OS was significantly correlated with pN2 (*P* = .031) and high HO‐1 levels (*P* = .029; Table 2). In addition, DFS was also significantly correlated with pN2 (*P* = .004; Table 2). However, in the multivariable analysis, none of the variables were independent prognostic factors (Table 2). The assumption of proportional hazards was verified graphically and the likelihood ratio test for the goodness of fit of the multivariate analysis model was statistically significant (OS; *P* = .047, DFS; *P* = .036).

**TABLE 2:** Univariate and multivariate analysis of 5‐year OS and DFS

		Univariate analysis (OS)		Multivariate analysis (OS)		Univariate analysis (DFS)		Multivariate analysis (DFS)	
Variables		HR (95% CI)	*P*	HR (95% CI)	*P*	HR (95% CI)	*P*	HR (95% CI)	*P*
Age	<65 versus 65≦	0.493 (0.173–1.410)	.187	0.694 (0.188–2.565)	.584	0.359 (0.114–1.137)	.082	0.373 (0.092–1.522)	.146
Gender	Male versus Female	5.919 (0.606–57.851)	.126	3.746 (0.254–55.272)	.336	4.999 (0.510–49.051)	.167	4.972 (0.326–75.831)	.249
pT	pT1‐2 versus pT3	2.263 (0.830–6.170)	.111	1.366 (0.460–4.053)	.575	1.624 (0.597–4.414)	.342	1.396 (0.476–4.091)	.543
pN	pN1 versus pN2	2.963 (1.104–7.948)	.031^a^	2.117 (0.601–7.461)	.243	4.988 (1.674–14.862)	.004^a^	3.055 (0.906–10.304)	.072
G	G1/2 versus G3	0.553 (0.193–1.580)	.269			0.556 (0.192–1.615)	.281		
Microlymphatic invasion	Absence versus Presence	1.556 (0.572–4.227)	.386			1.390 (0.503–3.836)	.525		
Microvascular invasion	Absence versus Presence	1.853 (0.680–5.050)	.228			2.500 (0.855–7.308)	.094		
NACRT efficacy(Primary tumor)	Grade 0‐1b versus Grade 2	0.438 (0.141–1.359)	.153			0.321 (0.090–1.142)	.079		
NACRT efficacy(LN metastasis)	LCRER versus HCRER	1.428 (0.527–3.868)	.484			2.336 (0.751–7.264)	.143		
Nrf2	Low versus High	1.635 (0.532–5.027)	.391			1.135 (0.393–3.278)	.815		
HO‐1	Low versus High	4.114 (1.157–14.626)	.029^a^	3.621 (0.955–13.731)	.059	2.754 (0.770–9.846)	.119		
8‐OHdG	Low versus High	1.142 (0.420–3.101)	.795			1.332 (0.483–3.678)	.580		
Ki‐67	Low versus High	1.548 (0.585–4.098)	.379			1.265 (0.468–3.414)	.643		

Abbreviations: DFS, disease‐free survival; HCRER, high chemoradiation therapy effective rate; LCRER, low chemoradiation therapy effective rate; LNM, lymph node metastasis; NACRT, neoadjuvant chemoradiation therapy; OS, overall survival.

a Statistical significance.

## 4 DISCUSSION

LN metastases are the most important poor prognostic factors for ESCC.^6^, ^7^ However, there is no standardized method for determining histologically defined therapeutic efficacy of NACRT on multiple LN metastases. This could explain why the TRG of pp‐MLNs varied in patients with multiple LN metastases.^8^ In this study, 40% of the clinically effective cases turned out to be histologically NACRT‐ineffective in primary tumors, indicating that the therapeutic response to NACRT could be different between primary tumors and LN metastases. Therefore, an accurate evaluation method to determine the therapeutic efficacy of NACRT in ESCC is warranted for appropriate clinical management of the patients.

Further, in this study, the histologically defined therapeutic effects of NACRT in LN metastases were tentatively classified according to CRER. Significant histologically determined treatment resistance to NACRT was also detected in those harboring high HO‐1 levels. The cytoprotective effects of HO‐1 are mediated via its metabolites and the subsequent formation of free iron that results in the production of the ferritin heavy chain, an iron‐chelating protein, and subsequent activation of Fe‐ATPase, allowing cytosolic iron efflux in carcinoma cells.^15^, ^27^ However, this reaction decreased intracellular Fe^2+^ and prevented the production of ROS via the Fenton reaction.^28^, ^29^ Therefore, high HO‐1 levels generally reduced ROS levels and could result in the development of therapeutic resistance to chemoradiation therapy. However, further investigations are required for clarification.

Both HO‐1 and Nrf2 were significantly lower in LN metastases than in primary tumors, which suggested that the antioxidant response to NACRT was weak in LN metastases. In addition, this may explain the favorable pathological response detected in LNs in our study. The combination of cisplatin‐based chemotherapy and radiation therapy is the main therapeutic approach for patients with ESCC, and the generation of ROS during and after this therapy is a prerequisite for the therapy's cytotoxic effect on carcinoma cells.^30^, ^31^ Therefore, the high expression of antioxidant enzymes caused by activating intracellular Nrf2 signaling pathways in carcinoma results in lower ROS levels and therapeutic resistance to chemoradiation treatment in ESCC. In addition, the Ki‐67 labeling index was also significantly lower in LN metastases than in primary tumors, suggesting a lower cell proliferation due to decreased Nrf2 expression in LN metastases.

The differences in the up‐regulation of the Nrf2 pathway between primary and metastatic lesions could be due to the intra‐tumoral heterogeneity of ESCC. Genomic alterations in the *Nrf2* pathway were enriched, resulting in high Nrf2 expression, particularly in the ESCC subtype mainly reported in East Asian patients.^32^ Patients with ESCC having LN metastases show high intra‐tumoral heterogeneity.^33^, ^34^ Therefore, down‐regulation of Nrf2 and antioxidant proteins derived from the Nrf2 pathway such as HO‐1 in LN metastases may have occurred due to the lower intra‐tumoral heterogeneity in LN metastases than in primary tumors. However, differences in intra‐tumoral heterogeneity between LN metastases and primary tumors of patients with ESCC are unknown and require further investigations. In addition, ESCC is known to be more likely to develop LN metastases earlier than other gastrointestinal tract cancers.^35^, ^36^ LN metastases in many of human malignancies including ESCC is also well‐known to be associated with epithelial‐mesenchymal transition (EMT) and mesenchymal‐epithelial transition (MET).^37^ Therefore, the tissue microenvironment between primary tumor and LN metastases could be altered during the process of EMT and/or MET and could have subsequently affected the upregulation of Nrf2 pathway. However, further investigations are required in this regard for clarification.

The survival analysis in this study indicated the potential clinical utility of evaluating the HO‐1 status in LN metastases when assessing the therapeutic outcome of patients with residual LN metastases receiving NACRT. Down‐regulation of the *HO‐1* gene via inhibition of the Nrf2 signaling pathway decreases radiation tolerance.^38^ HO‐1 is also associated with cisplatin resistance in various cancer types.^39^, ^40^ Therefore, high HO‐1 levels in LN metastases can be used to assess the clinical outcomes of patients treated with chemotherapy or chemoradiation therapy regimens, including cisplatin. However, DFS did not vary significantly with HO‐1 levels in LN metastases. Furthermore, in contrast to high HO‐1 levels in primary tumors, HO‐1 levels in LN metastases were not necessarily correlated with clinical outcomes of the patients. In addition, multivariable analysis revealed that no marker was an independent predictive factor of clinical outcomes. This discrepancy may be due to the intrinsic association among the multiple factors in this study. For example, an association between HO‐1 and phosphorylated PTEN deletion mutants has been reported; this association is responsible for the invasiveness of carcinoma cells and metastasis by angiogenesis promotion.^41^, ^42^ These factors can possibly account for the correlation between high HO‐1 levels and established clinicopathological variables, including pT and pN. In addition, the small sample size with insufficient events per variable is also considered another reason for the discrepancy.^43^

Results of this study revealed that the enhancement of the Nrf2 signaling pathway was less in LN metastases than in primary tumors, which also indicated that NACRT contributed to favorable clinical outcomes and prognosis of patients with ESCC having LN metastases. This study had some limitations. First, this was a retrospective, single‐institution study with a small sample size. The standard neoadjuvant therapy for ESCC is NAC, and not NACRT. Accordingly, NACRT has not been administered in our institution since 2015. Therefore, our results could not be validated with larger cohorts in our institution. In addition, the small sample size may have influenced the results of the multivariate analysis. However, our results reveal a significant correlation between HO‐1 level and OS or therapeutic efficacy of NACRT in LN metastases, despite the relatively small sample size. Second, immunoreactivity was assessed at the LN with the largest residual tumor volume; however, there are no established methods for determining the histological efficacy of preoperative therapy in LN metastases. Third, the changes of protein expression in some cases in which carcinoma cells were not discernible following NACRT could not be evaluated. Lastly, none of the factors examined in the multivariate analysis could independently predict clinical outcomes. This may be due to the intrinsic correlations among them. Therefore, further validation studies with larger cohorts are warranted to clarify the clinicopathological significance of these results.

## 5 CONCLUSION

Enhancement of the Nrf2 signaling pathway was less in LN metastases than in primary lesions. High HO‐1 levels in LN metastases may be a prognostic marker for OS in patients with ESCC receiving NACRT.

## CONFLICT OF INTEREST

The authors declare that they have no conflict of interests.

## AUTHOR CONTRIBUTIONS

All authors had full access to the data in the study and take responsibility for the integrity of the data and the accuracy of the data analysis. *Conceptualization*, R.A., F.F., T.F., T.K., H.S.; *Methodology*, R.A., F.F., T.N., N.N., H.S.; *Investigation*, R.A., J.T., T.Y.; *Formal Analysis*, R.A., F.F., H.I.; *Writing—Original Draft*, R.A., F.F., H.I., H.S.; *Writing—Review & Editing*, R.A., F.F., H.I., H.S.; *Visualization*, R.A.; *Supervision*, F.F., H.I., Y.G., S.U., T.F., H.O., K.T., C.S., Y.T., T.H., H.S.; *Funding Acquisition*, T.F., T.K.; *Data Curation*, R.A.; *Project Administration*, F.F., T.F., T.K., H.S.

## ETHICAL STATEMENT

Ethics approval and consent to participate 102 The study protocol was approved by the Ethics Committee of the Tohoku University 103 School of Medicine (Accession No. 2020‐1‐87), and informed consent was obtained from 104 all patients prior to surgery through signed consent forms.

## Supporting information


**Supplementary Table S1** Features of the primary antibodies used in immunostaining of this study.

**Supplementary Table S2** Tumor regression grade of the plausible positive metastatic lymph nodes and effectiveness of chemoradiation therapy in the study participants.

**Supplementary Table S3** Clinicopathological features of participants.

## Data Availability

The datasets used and/or analyzed during the current study are available from the Supplementary Material and from the corresponding author upon reasonable request.
